# High speed functional imaging with source localized multifocal two-photon microscopy

**DOI:** 10.1364/BOE.9.003678

**Published:** 2018-07-12

**Authors:** Peter Quicke, Stephanie Reynolds, Mark Neil, Thomas Knöpfel, Simon R. Schultz, Amanda J. Foust

**Affiliations:** 1 Department of Bioengineering, Imperial College London, SW7 2AZ, UK; 2 Centre for Neurotechnology, Imperial College London, SW7 2AZ, UK; 3 Department of Electrical and Electronic Engineering, Imperial College London, SW7 2AZ, UK; 4 Department of Physics, Imperial College London, SW7 2AZ, UK; 5 Department of Medicine, Imperial College London, SW7 2AZ, UK

**Keywords:** (100.0100) Image processing, (110.0110) Imaging systems, (180.0180) Microscopy

## Abstract

Multifocal two-photon microscopy (MTPM) increases imaging speed over single-focus scanning by parallelizing fluorescence excitation. The imaged fluorescence’s susceptibility to crosstalk, however, severely degrades contrast in scattering tissue. Here we present a source-localized MTPM scheme optimized for high speed functional fluorescence imaging in scattering mammalian brain tissue. A rastered line array of beamlets excites fluorescence imaged with a complementary metal-oxide-semiconductor (CMOS) camera. We mitigate scattering-induced crosstalk by temporally oversampling the rastered image, generating grouped images with structured illumination, and applying Richardson-Lucy deconvolution to reassign scattered photons. Single images are then retrieved with a maximum intensity projection through the deconvolved image groups. This method increased image contrast at depths up to 112 μm in scattering brain tissue and reduced functional crosstalk between pixels during neuronal calcium imaging. Source-localization did not affect signal-to-noise ratio (SNR) in densely labeled tissue under our experimental conditions. SNR decreased at low frame rates in sparsely labeled tissue, with no effect at frame rates above 50 Hz. Our non-descanned source-localized MTPM system enables high SNR, 100 Hz capture of fluorescence transients in scattering brain, increasing the scope of MTPM to faster and smaller functional signals.

## 1. Introduction

Two-photon laser scanning microscopy (2PLSM, [1]) is widely used in neuroscience due to its ability to image cellular and subcellular structures at high spatial resolution with low phototoxicity and photobleaching. In combination with intracellular calcium indicators, it allows readout of neuronal action potentials (APs) from single cells in highly scattering mammalian brain tissue. The inherent optical sectioning from nonlinear fluorescence excitation also enables the discrimination of different activity amongst densely packed neurons.

As a laser scanning technique, 2PLSM has to trade off temporal resolution, spatial sampling and fluorescence excitation. Resonant galvanometers, improved scanning strategies [2], point spread function sculpting [3] and temporal multiplexing [4] offer improved temporal performance. In its basic implementation, however, frame rates for most neuroscience applications are limited to around 10 Hz for fields of view (FOV) of up to 500 × 500 μm. This is adequate to detect slow calcium transients if low temporal precision on the inference of the underlying AP timing is sufficient. However, there is evidence that AP timing and frequency play a crucial role in sensory encoding and processing [5], and increasing imaging temporal resolution whilst maintaining signal-to-noise ratio (SNR) decreases the error in AP timing estimation [2, 6]. Secondly, the faster dynamics and smaller size of signals such as membrane potential or neurotransmitter release and reuptake limit standard 2PLSM’s applicability to these signals.

Spatial multiplexing has been widely explored as a strategy to improve two-photon imaging’s temporal resolution without sacrificing pixel dwell time and fluorescence excitation [7]. Digital holography allows up to 100% duty cycles by shaping light only over regions of interest, but requires preselecting specific structures and ignoring others [8–15]. Multifocal two-photon microscopy (MTPM) parallelizes two-photon acquisitions by scanning multiple beamlets and allows dense sampling without target preselection. Multifocal microscopes have been implemented using microlens arrays [16], diffractive optical elements [17], etalons [18], beamsplitters and mirrors [19], and spatial light modulators [20]. Longer pixel dwell times and therefore total excited photon flux can be achieved with MTPM without reducing the frame rate. This increases Poisson-noise limited SNR compared to single-spot two-photon for equivalent imaging speeds, with SNR scaling as $\sqrt{N}\cdot {\text{SNR}}_{0}$, for *N* additional equal foci and a single spot SNR of SNR_0_. This has been exploited to image calcium in neural cells with high SNR [21–23]. Spatial multiplexing of two-photon excitation, however, removes the ability to assign excited fluorescence to a single spatial location with total certainty. The imaged fluorescence is susceptible to crosstalk, degrading contrast in highly scattering tissue such as the mammalian brain. This limits achievable imaging depths and mixes functional signals from different cells, confusing analysis of their underlying activity. Previous MTPM implementations improved robustness to scattering by descanning fluorescence onto multianode photomultiplier tubes (PMTs) [24] and developed reassignment algorithms for photons collected on the wrong anode [25].

The descanned collection, however, reduces photon collection efficiency due to the additional collection optics by 15–50% and, even if non-descanned [26], must use relatively low quantum efficiency (QE) multianode PMTs (∼16% QE at 550 nm (H7546, Hamamatsu)) compared to the sCMOS cameras used in wide-field detection (∼82% QE at 550 nm (Orca Flash 4.0 V2, Hamamatsu)), decreasing functional SNR.

We have developed a novel photon source localization and MTPM strategy implemented with non descanned epifluorescent collection for fast functional imaging of neural signals. This has allowed us to maintain the increased SNR of MTPM whilst mitigating the effects of scattering on the recording. In this paper, we describe our MTPM implementation and algorithm and show that it increased image contrast at depth and reduced functional crosstalk between pixels in neural imaging data. We also analyzed the effect of source localization on the SNR and found it was maintained for densely labeled samples.

## 2. Methods

### 2.1. Multifocal two-photon setup

**Fig. 1:**
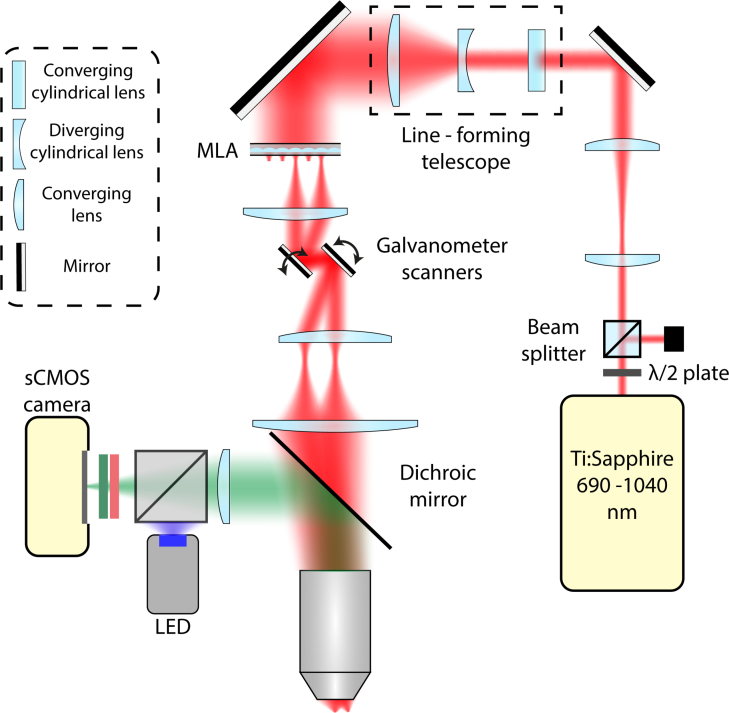
The multifocal apparatus. The laser beam is shaped into a line by an asymmetric telescope before passing through a microlens array which splits it into multiple beamlets. Individual beamlets are collimated and directed onto galvanometer mirrors which are conjugate with the back focal plane of the objective lens. The beamlets are raster scanned in the sample forming an image which is collected onto a CMOS camera synchronised with the galvanometers. A wide-field LED excitation path is used for comparison with the multifocal excitation.

**Fig. 2:**
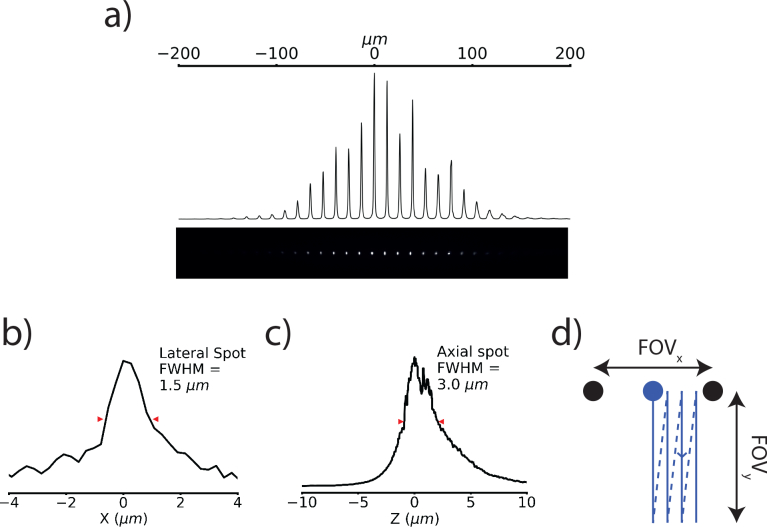
Optical characteristics of the multifocal array. a) Example image of a line beamlet array on a thin fluorescent slide. b) Lateral and c) axial cross sections of a single beamlet scanned through a thin fluorescent layer. d) An illustration of the scanning strategy used. Each beamlet is rastered in a rectangle orthogonal to the beamlet axis to build up the image. The temporally oversampled images used in the source localization process consist of one image per vertical raster line, in this illustration, four images.

We built a custom MTPM for functional neural imaging (Fig. 1

). A Ti:Sapphire laser beam (Mai Tai HP, Spectra Physics), tuned at 800 nm with a 80 MHz repetition rate, passed through a half wave plate and polarising beam cube to control the power, typically between 400 and 700 mW at the sample. A 6:5 Keplerian telescope (focal lengths +300 mm, Thorlabs AC508-300-B; and +250 mm, Edmund Optics G322 311 525) reduced and relayed the beam waist to the downstream optics. A second telescope consisting of a +700 mm cylindrical lens, a −20 mm cylindrical lens, and a + 50 mm spherical lens (Thorlabs, LJ1836L1-B, LK1085L1-B and LA1131-B) shaped the beam into a line before it entered a micro lens array (MLA, 0.15 mm pitch, 0.26 mm focal length, Ultra Precision and Structured Surfaces (UPS2)) which split it into beamlets. Sandwiching the MLA between two 1″ glycerol-filled glass coverslips increased its focal length to +0.975 mm to achieve the desired objective fill fraction of 0.8. The shape of the beam into the MLA determined the envelope of the beamlet line array in the sample (Fig. 2(a)

). Each beamlet rastered a rectangle in the sample, with the rectangle’s long axis perpendicular to the beamlet line’s axis (see Visualization 1 and Fig. 2(d)). This allowed efficient utilization of the CMOS camera’s central section and therefore maximized the frame rate. It is important that the line lie centrally on a single line of microlenses to achieve maximum excitation efficiency in the sample. A +20 mm lens (Thorlabs, LA1074-B) collimated the beamlets into galvanometer mirrors (Cambridge Technology) before they passed through the scan (+75 mm, Thorlabs AC254-075-B), tube (+300 mm, Thorlabs AC508-300-B) and objective (1.0 NA, 25×, Olympus XLPlan N) lenses into the sample. A 670 nm dichroic (Chroma, ZT670rdc) directed non-descanned epifluorescence through a +180 mm tube lens (Thorlabs, AC508-180-A) onto the CMOS camera (Orca Flash 4.0 V2, Hamamatsu). We filtered emission with 525/50 nm bandpass (Chroma, ET 525/50) and 750 nm short pass (Semrock, FF01-750/SP-25) filters. A blue light-emitting diode (LED; Thorlabs M490L3, excitation filter Semrock FITC-Ex01-Clin-25), collimated with a 16 mm focal length aspheric lens (Thorlabs ACL25416U-A) and reflected to the sample with a long pass dichroic mirror (Semrock FF495-DI03-25X36), excited one-photon epifluorescence in wide-field mode.

**Fig. 3:**
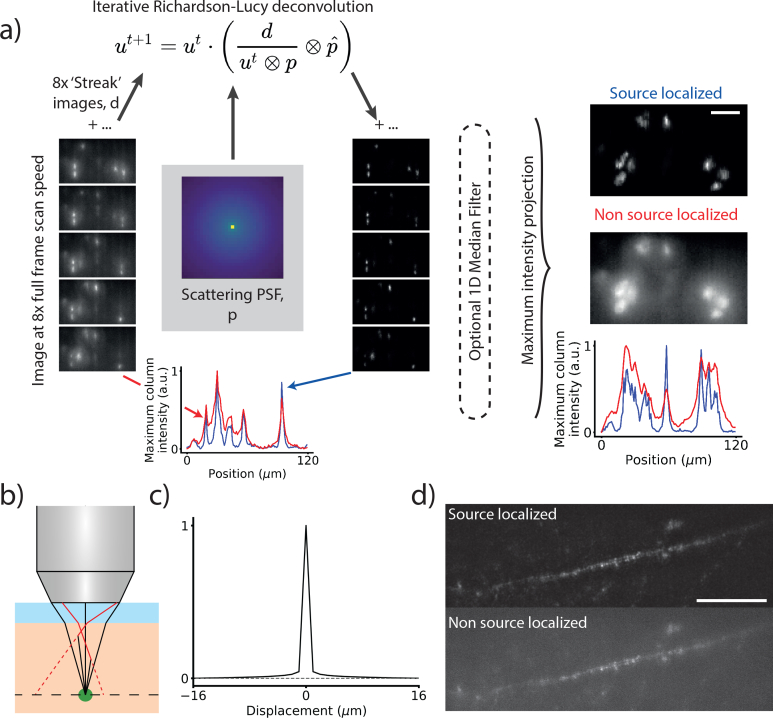
The source localization process. a) To generate a single source-localized frame, eight temporally oversampled images are acquired, corresponding to a single line scan from each beamlet. A subset of these are shown in the leftmost column of ‘streak’ images. Any illumination arriving onto the camera not on the line has arisen from scattering of the emission. We use Richardson-Lucy deconvolution to reassign the light back to its probable origin. The right column of ‘streak’ images shows the deconvolved images. Below these columns is a maximum intensity projection in *y* of the bottom ‘streak’ image showing the increase in contrast due to source localization. Once the light is reassigned, we take a maximum intensity projection through the temporally oversampled frames to recover a single frame. The two right-most images show a comparison between a source localized frame and an unprocessed image and below them a maximum intensity projection in *y* to show the increased contrast from source localization. Scale bar 20 μm. b) A diagram of the Monte Carlo method used to generate a deconvolution kernel for the Richardson-Lucy deconvolution. Photons from a point source (green dot) were propagated through a scattering medium and backprojected when they reached the surface. Red rays were scattered and the dotted lines show backprojection to their apparent origin. Black rays are unscattered light. c) A cross section through the scattering PSF used for the deconvolution. The sampled PSF was approximately radially symmetric. d) A source localized (top) and non-source localized (bottom) image of a layer 2/3 pyramidal cell’s apical dendrite labeled with membrane-targeted GFP. 12 lines per frame, scale bar 50 μm, see Visualization 1.)

A National Instruments DAQ (PCIe-6321) and custom LabVIEW code controlled the galvanometers, and Micromanager [27] controlled the camera acquisition. Spike2 electrophysiology software and a Power1401 digitizer (Cambridge Electronic Design) controlled the excitation shutter, electrophysiological stimuli and master timing of the galvanometers scans and camera acquisitions. To acquire a synchronous imaging and electrophysiology run, the digitizer triggered the camera in ‘free run’ mode at the beginning of each acquisition. The digitizer recorded the camera readout start signal and triggered each full frame scan of the galvanometer mirrors. For functional imaging, we rastered each beamlet in a rectangle with 8 lines filling the beamlet pitch to fully cover the field of view. An illustration of an equivalent 4-line scan pattern is shown in Fig. 2(d). For a standard image, we set the exposure time to just over the scan time. As the beamlets have an approximately Gaussian envelope (Fig. 2(a)), the resulting image has a Gaussian intensity profile parallel to the beamlet axis. To apply the source localization process, we imaged at approximately 8 times this frame rate so that in each frame a single line from each beamlet was imaged (i.e. one image per vertical line in Fig. 2(d), example images shown in Fig. 3(a)

). The exposure time of the camera was adjusted to synchronize it with the galvanometers. All analysis and image processing was carried out using Python 3 and results are reported as mean ± standard error of the mean.

### 2.2. Image reconstruction

To improve MTPM’s resistance to scattering, we developed a source-localization process to reassign scattered photons to their likely original emitted locations. Instead of exposing the camera for the full 8 line scan pattern, we imaged at 8× the scan speed to generate a set of 8 ‘streak’ images per time point, one for each line (see Fig. 2(d) for equivalent 4-line diagram, example ‘streak’ images in Fig. 3(a) and full image build-up in Visualization 1). Taking the mean of these frames is equivalent to exposing the camera for a full scan. Due to the structured illumination in each ‘streak’ image, any light collected off the streaks is known to be scattered. A 100-iteration Richardson-Lucy deconvolution ([28, 29], implemented in scikit-image [30]), estimated the emitter distribution in the absence of scattering, using a deconvolution kernel derived from Monte Carlo modeling of photon propagation in scattering tissue. We initialized the deconvolution with a flat prior and updated the image using the equation

(1) $$u_{t+1}=u_{t}\cdot \left(\frac{d}{u_{t}⊗p}⊗\hat{p}\right)\;,$$

where *u* is the deconvolved image, *t* the current iteration number, *d* the measured image, *p* the point spread function and *p̂* the point spread function mirrored along the *x* = *y* axis. Reflectively padding the boundaries reduced edge artefacts in the final image.

We generated the PSF using Monte Carlo simulation in a similar manner to [31] by simulating the propagation of photons from a point source at (*x*, *y*, *z*) = 0 through a slab of scattering tissue extending from 0 ≤ *z* ≤ *z_h_*. We simulated photons with an initial propagation angle drawn from a uniform distribution over the elevation and azimuthal angles corresponding to a NA of 1 to match the objective used in this experiment. The contribution from photons outside of this range was assumed to be negligible. The photons were propagated through the tissue for a length drawn from an exponential distribution with a characteristic scattering length *μ* = 20 mm^−1^ [31]. If *z* < *z_h_*, we simulated a scattering event by drawing a new propagation angle from a uniform distribution between 0 and 2*π* in azimuth and from a Henyey-Greenstein phase function [32] with a forward scattering anisotropy of *g* = 0.9 in elevation [31]. The propagation and scattering steps were then repeated until the photon left the tissue. The photon was then backprojected along its final propagation angle to find its (*x*, *y*) position for *z* = 0 (Fig. 3(b)). We assigned the photon to a camera pixel according to its location and repeated the process for 10^8^ photons to sample the kernel. This analysis did not consider the effect of the PSF of the microscope upon the deconvolution.

Multiple PSFs were generated for different modeled depths and applied to test data to decide upon a final PSF and iteration number to use in the reconstructions. We manually reviewed the contrast and high spatial frequency content of a test image after deconvolution with different PSFs and found the best results when using a scattering kernel for a tissue depth of *z_h_* = 190 μm, although good results were observed for depths of 100 to 200 μm, the maximum depth modeled. We show a cross section through the PSF in Fig. 3(c). Although the same deconvolution parameters were applied to all data for comparison, tailoring the PSF and deconvolution parameters to an acquisition’s specific parameters could improve performance. Deconvolving the streak images suppressed off-streak illumination while increasing the brightness of the illuminated features on the line as shown in Fig. 3(a). For some deeper, lower contrast images a one-dimensional median filter parallel to the streaks was applied after the deconvolution to suppress noise and improve contrast. Complete frames for each time point were then recovered by taking a maximum intensity projection through each set of 8 deconvolved streak images.

### 2.3. Analysis of depth penetration

To determine whether source localization reduced the effects of scattering at depth in brain tissue, we took depth stacks through fixed brain slices containing cells expressing membrane localized GFP. We used the Michelson contrast metric [33] to measure the relative contrast of the cells and their background, defined as

(2) $$C=\frac{I_{\text{max}}-I_{\text{min}}}{I_{\text{max}}+I_{\text{min}}},$$

where *C* is the contrast, *I_max_* is the maximum pixel intensity of a cell and *I_min_* is the minimum intensity of the cell’s surroundings, the background. Scattering will tend to increase the intensity of the background and decrease the intensity of the cell, reducing this contrast metric. In order to correctly identify *I_max_* and *I_min_* as cellular GFP signal and extracellular space and not from, e.g., uneven illumination, we segmented the cells and their surrounds from the image and measured the intensities from these areas. To segment the cells we thresholded the image, choosing the pixels with intensities above the 80% cutoff of the maximum of the image histogram and removed small objects by 4 binary erosion iterations followed by 4 dilation iterations. The contrast was set to zero if all or no pixels were segmented. We calculated *I_max_* and *I_min_* as the 99.9th percentile and 10th percentile of the segmented intensities respectively. The metric was robust to changes in the percentiles used.

### 2.4. Modeling the effect of foci separation on contrast at depth

To assess the effect of varying the foci separation on the source localization process we modeled the contrast reduction with depth for different spacings and tissue scattering coefficients. We used tissue scattering values of *μ* = 10, 20 and 40 mm^−1^. We split a reference single-spot two-photon image of GFP-expressing cells into sets of images consisting of vertical lines analogous to the ‘streak’ images in Fig. 3(a), using a different streak separation for each set. These are equivalent to the temporally oversampled images used in the source localization process with the oversampling factor required the line separation in pixels. We convolved these images with scattering kernels for different depths and scattering coefficients generated using our Monte-Carlo model. Noise was added by replacing each pixel intensity value, *I*_0_, with a value drawn from a Gaussian distribution, *P*(*I* | *μ*, *σ*), with *μ* = *I*_0_ and *σ* = *I*_0_/10. To generate a set of source localized images we applied Richardson-Lucy deconvolution to the noisy line image series using the scattering kernels we used previously cropped to 32 × 32 pixels. We then generated our final images for comparing source-localized and standard MTPM by taking a sum projection through the noisy line images and a maximum intensity projection through the deconvolved images, respectively. For each line separation and scattering coefficient we estimated the expected 50% contrast cutoff depth by fitting a 3^rd^ order polynomial to the contrast depth profile.

### 2.5. Preparation of brain slices for functional imaging

To test our multifocal setup and source localization algorithm on time-resolved image series, we collected neuronal data from mouse acute brain slices. All animal experiments were performed under institutional guidelines, were approved by the Home Office (UK) and were in accordance with the UK Animals (Scientific Procedures) Act 1986 and associated guidelines. We used two different preparations: slices where many cells were labeled with bath application of an acetoxymethyl-ester (AM-ester) calcium dye (referred to as multi-cell labeling), and slices where a single cell was intracellularly loaded with calcium dye potassium salt (single-cell labeling).

Slices from mice aged between 28 and 42 days old were prepared for calcium imaging using the protective recovery method (www.brainslicemethods.com, [34]). Mice were anaesthetised with isoflurane and decapitated before their brains were quickly removed and placed into ice-cold artificial cerebro-spinal fluid (Na-ACSF) containing (in mM): 125 NaCl, 25 NaHCO_3_, 20 glucose, 2.5 KCl, 1.25 H_2_PO_4_, 2 MgCl_2_, 2 CaCl_2_ for multi-cell labeling; and 124 NaCl, 2.5 KCl, 1.2 NaH_2_PO_4_, 24 NaHCO_3_, 5 HEPES, 12.5 Glucose, 2 MgCl_2_, 2 CaCl_2_ for single-cell labeling. The solution was diluted to between 300 and 310 mOsm/kg, pH adjusted to 7.3 – 7.4 with NaOH, and oxygenated with 95% O_2_/5% CO_2_. 400 μm slices were cut using a Campden Microtome 7000 and placed for 10 – 15 minutes into NMDG-ACSF containing: (in mM) 110 N-Methyl-D-glucamine, 2.5 KCl, 1.2 NaH_2_PO_4_, 25 NaHCO_3_, 25 Glucose, 10 MgCl_2_, 0.5 CaCl_2_, adjusted to 300 – 310 mOsm/kg, pH 7.3 – 7.4 with HCl, oxygenated with 95% O_2_/5% CO_2_ at 34 °C before being transferred back into the original sodium-containing ACSF. This short ‘protective recovery’ step increases the number of healthy cells in brain slices [34]. The slices then rested at room temperature for an hour before multi-cell labeling with Cal-520 AM-ester dye or were left until needed for single-cell labeling. For multi-cell labeling 50 μg of Cal-520 AM ([35], AAT-Bioquest) was dissolved in 10 μl of DMSO with 10% w/v Pluronic F-127 (Invitrogen) and 0.5% v/v Kolliphor EL (Sigma-Aldrich) [36]. The slices were then incubated for 40 minutes at 34 °C in 2 ml of Na-ACSF with the Cal-520 AM/DMSO mixture pipetted onto the surface of each slice. The 2 ml of Na-ACSF was kept oxygenated by blowing 95% O_2_/5% CO_2_ onto its surface. After loading, the slices rested in Na-ACSF for at least 20 minutes before recordings were taken.

We recorded single cell responses to excitatory stimulation for both multi-cell and single-cell labeled slices. For multi-cell labeled slices, a concentric bipolar electrode was placed at the cortical layer 6 and white matter boundary to extracellularly stimulate for 0.5 ms at 1 Hz. Responding cells were found by imaging in nearby areas of cortical layers 2/3 and 5. We adjusted the current delivered to the threshold required to evoke an AP-induced calcium transient. Imaging trials were performed at 20 and 50 Hz using MTPM and LED illumination.

To image single-cell labelled cells, cortical cells were patched using 4 – 7 MOhm patch pipettes containing intracellular solution consisting of (in mM) 130 K-Gluconate, 7 KCl, 4 ATP - Mg, 0.3 GTP - Na, 10 Phosphocreatine - Na, 10 HEPES, 0.1 Cal-520 potassium salt (AAT-Bioquest). Trials were rejected when resting membrane potential exceeded −60 mV with zero injected current. After sealing and breaking in, the calcium dye diffused into the cell for 30 minutes while access resistance was kept below 15 MOhm by suction. After intracellular loading, imaging trials were taken at 10, 20, 50, 100 and 200 Hz using MTPM and LED illumination for two different trial types. The first trial consisted of eliciting pairs of APs with differing time separation. Six pairs of spikes were stimulated with 0.5 ms current injections with 0.3, 0.2, 0.1, 0.05, 0.02 and 0.01 s between the start of each stimulus and 6 s between the start of each pair. The second trial used 0.5 ms current injections to elicit trains of spikes with identical inter stimulus intervals. 1, 3, 5, 7 and 9 spikes were elicited with 0.05 s between stimuli and 6 s between trains.

All MTPM imaging trials were taken at room temperature with a power under the objective of 400 – 700 mW. Imaging cells during whole-cell patch clamp recordings did not affect resting membrane potential, input resistance or cell capacitance at these power levels. Trial order was generated randomly for each cell to control for the effects of photobleaching and physiological rundown.

### 2.6. Analysis of calcium imaging videos

#### 2.6.1. Pixel crosstalk

To determine the effect of the source localization algorithm on functional neural imaging data, we first tested whether source localization would reduce the crosstalk between the fluorescent signals from different cells caused by scattering. To quantify this, we selected regions of interest (ROIs) containing a single responsive cell and quantified the contribution of the intracellular functional time course to the background in the surrounding pixels. If this contribution was high, then time courses from other cells in the ROI would be contaminated by scattered light from the central cell. We analyzed both multi-cell and single-cell labeled data. While the technique is optimized for multi-cell imaging, the single-cell image series enable discernment between fluorescence arising from network activity and fluorescence scattered from a single, active cell.

For multi-cell labeling, cells responsive to stimulation surrounded by non-responsive cells were identified by hand and ∼ 30 × 30 μm regions of interest (ROIs) selected for the analysis. We segmented the intracellular ROIs from the source-localized video series using local correlation maps. We generated a 2D image by calculating the Pearson correlation of every pixel with its 8-connected neighbors. This was then thresholded using the method in [37]. Binary closing and 2-round opening was applied to remove smaller objects. If more than one region remained, the largest connected component was chosen as the intracellular ROI. For each cell, we chose a mean segmentation by combining the segmentation results from individual video series and choosing pixels that were included in more than a quarter of them. Intracellular time courses were extracted from MTPM, source-localized MTPM, and LED image series by taking the mean values of the cellular ROIs generated using the source-localized MTPM. Extracellular traces were generated by averaging the pixels in the surrounding area, not including the cellular ROIs. We generated ΔF/F_0_ traces by dividing the time courses by the mean of the baseline brightness minus the average dark frame value. Intracellular calcium concentration dynamics can be inferred from calcium indicator ΔF/F_0_ traces. We measured the signal localization due to the source localization algorithm as the average size of the ratio between the intracellular ROI calcium transient and the extracellular ROI calcium transient, which we called the signal localization ratio (SLR).

Segmenting the single-cell labeled data was simpler due to the lack of background staining. We segmented the cells by generating and thresholding a 2D variance map of the pixel time courses and similarly removed small objects by binary closing and opening. We calculated the extracellular ROI by extending for 5 pixels in *x* and *y* around the bounds of the segmented cell to avoid a ringing artefact from the deconvolution in the single-cell labeled images where there was a small anticorrelated signal. This artefact is due to the highly localized single-cell labeling and would not occur in densely labeled tissue due to the maximum projection step in the reconstruction. To avoid overestimating the power of our technique we used this smaller ROI and counted anticorrelated ringing as unreassigned signal. We carried out the same analysis as for extracellularly loaded data, excluding signal from the dye inside the patch pipette.

#### 2.6.2. Signal-to-noise ratio

We examined whether the reconstruction process affected the SNR of the functional time courses as noise amplification can be a side effect of Richardson-Lucy deconvolution. For intracellular data for which we acquired simultaneous whole-cell patch clamp electrophysiology, we estimated the single-spike signal amplitude by fitting a model of the indicator dynamics. We model a fluorescence signal consisting of *K* spikes at times ${\left\{t_{k}\right\}}_{k=1}^{K}$ as

(3) $$f(t)=\sum_{k=1}^{K}A\;c_{\alpha ,\gamma }\;\left(e^{-\alpha (t-t_{k})}-e^{-\gamma (t-t_{k})}\right)\;u(t-t_{k}),$$

where *u*(·) is the indicator or step function, which is equal to zero for negative arguments and one otherwise, and the parameters {*A*, *α*, *γ*, *c*_*α*,*γ*_} define the shape of the calcium transient. The speed of the pulse’s rise and decay is determined by the parameters *α* and *γ*, which were estimated from [35] to be 3.18 s^−1^ and 34.39 s^−1^ respectively. The factor *c*_*α*,*γ*_ is a normalization constant, which ensures that the peak amplitude of a calcium transient is at *A*. The signal samples, *y*[*n*], are assumed to be corrupted by additive noise in the acquisition process, such that *y*[*n*] = *f*[*n*] + *∊*[*n*], where *f*[*n*] = *f*(*nT*) are samples of the fluorescence signal with time resolution *T* and *∊*[*n*] are samples from a zero-mean normal distribution with standard deviation *σ*. The noise level of each trace, *σ*, was estimated from the sample standard deviation of the final 0.2 s of the trace in which there was no spiking activity. Given knowledge of the spike timings from the electrophysiology and the signal samples, the amplitude parameter, *A*, was fit to Eq. (3) using a linear least squares program. For the extracellularly loaded data we were unable to fit a model as we did not have simultaneous ‘ground truth’ electrophysiology and so we estimated the signal size, *A*, as the peak intensity in a short window after the stimulus was applied. We measured the trace noise level by taking the standard deviation of the final 0.3 s of the trace when there was no activity. The peak signal-to-noise ratio (PSNR) of the data is then calculated as *A*^2^/*σ*^2^.

## 3. Results

### 3.1. Source localization increases contrast at depth

**Fig. 4:**
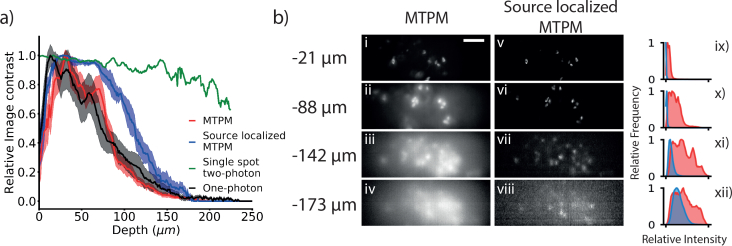
Characterization of the increased depth penetration from source localization. a) Image contrast plotted against depth from the tissue surface for one-photon wide-field LED illumination, MTPM, source localized MTPM and two-photon point scanning. Plotted as mean ± s.e.m. b) Example frames taken at different depths in fixed brain tissue without (i – iv) and with (v – viii) source localization. To the right ix) – xii) are the histograms of the normalized images, in red without source localization and blue with source localization. Scale bar 50 μm

Source localization decreases blur from scattering at depth in tissue. We took depth stacks through fixed cortical tissue containing cells labeled with GFP to determine whether our source localization procedure increased image contrast at depth. We calculated the changing contrast with depth for 5 different cortical fields of view for wide-field LED illumination, MTPM and source localized MTPM, (Fig. 4

), along with a single area for a typical 2PLSM for comparison. The data shows a drop off of contrast quickly with depth for all 3 modalities compared to a standard 2PLSM, as expected. The 50% cutoff for the source localized case of 112 ± 5 μm is significantly deeper than for the non source-localized (80 ± 3 μm, p = 0.008, paired T-test, n = 5 areas, mouse age 42 days) or LED illumination (72 ± 6 μm, p = 0.007, Welch’s two sample T-test, n = 5 areas, mouse age 42 days).

**Fig. 5:**
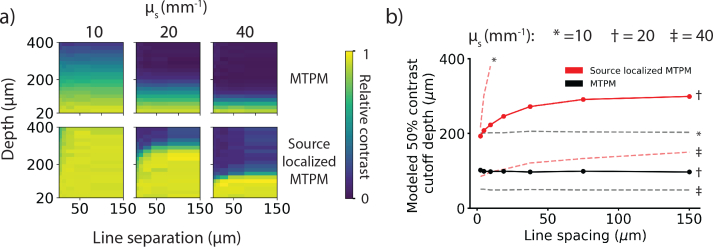
The predicted effect of changing foci separation on depth penetration. a) False color plots of relative contrast modeled at different depths for varying line spacing for standard MTPM and source localized MTPM for three different scattering coefficients b) A plot of the depths corresponding to a 50% decrease in relative contrast for MTPM (black) and source localized MTPM (red) for different line spacings. Solid lines show the data for the scattering coefficient used in the reconstructions of the experimental data, while dashed lines were modeled using different scattering coefficients. Source localization increases maximum imaging depth compared to standard MTPM, improving contrast more at larger line separations. Increased depth penetration must be traded off against increased imaging speed or SNR.

To investigate how changing the spacing between the foci might affect the depth penetration of source localized MTPM, we modeled the contrast loss with depth for foci separations from 2.3 – 150 μm for three different scattering coefficients between 10 and 40 mm^−1^. Smaller line separations allow for greater parallelization of the acquisition and therefore an increase in SNR for the same imaging speed. As shown in Fig. 5(a) & (b)

, source localization increases the maximum imaging depth compared to standard MTPM for all scattering coefficients and line separations. Source localization’s depth penetration increases asymptotically relative to standard MTPM with increasing line spacing. The asymptote value and rate of approach decrease with increasing scattering coefficient. At small line separation the deconvolution is less effective at reassigning scattered photons to their correct location. At the line separation implemented in this paper and expected experimental scattering coefficient (12.5 μm and 20 mm^−1^ respectively) our model predicts a 50% contrast cutoff at 98 μm for MTPM and 230 μm for source localized MTPM. These cutoffs are deeper than those found experimentally. This could be due to the modeling of the scattering medium as a homogeneous block, or an underestimation of the scattering coefficient of the tissue.

### 3.2. Source localization decreases functional crosstalk

**Fig. 6:**
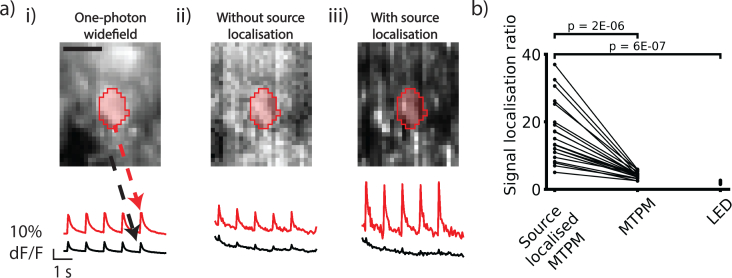
Source localization decreases the functional crosstalk between adjacent neuronal structures. a) Example ROIs and fluorescence time courses of multi-cell labeled neuronal tissue with a single responsive cell using i) wide-field LED, ii) MTPM and iii) source localized MTPM. Red traces are the average of the red intracellular region and black traces of the entire region outside the red area. In both the wide-field LED and MTPM case there is a significant component of the intracellular time course contaminating the extracellular pixels. This leads to difficulty discriminating between cells in areas with multiple active cells. Source localization drastically reduces the relative contribution of the intracellular time course to the extracellular region. b) Summary data showing the ratio of peak intracellular to extracellular response to excitatory stimulus (the signal localization ratio) over 20 trials. This is significantly higher for source localized MTPM compared to MTPM or wide-field LED illumination. Scale bar 10 μm.

Source localization reduces spurious functional signals from scattered light. Scattered in and out of focus light impedes single-cell resolution wide-field functional imaging in densely labeled samples. It is difficult to demix time courses from different cells, especially when the activity has a low SNR or is spatiotemporally dense. To test whether our source localization strategy reduced the contamination of extracellular pixels with functional signal, we compared the ratios of peak ΔF/F_0_ signal sizes from the mean time courses of segmented intracellular regions to the mean time courses of surrounding extracellular areas, the signal localization ratio (SLR, Fig. 6(a)

). A larger ratio indicates a smaller contamination of the extracellular pixels. Comparing the same raw data with and without source localization shows a significant increase in the SLR when source localization is applied, with the mean SLR increasing from 4.2 ± 0.2 to 16.9 ± 2.0 (p = 2E-06, paired t-test, n = 20 trials, mouse age 36 days, power used 700 mW, Fig. 6(b)). The SLR for the LED illumination in equivalent trials was found to be 2.03 ± 0.08, significantly lower than the source localized MTPM (p = 6E-07, Welch’s two sample T-test, n = 18 trials, mouse age 36 days).

To ensure that these results were not confounded by neuropil or multicellular signal excited by the field stimulation, we repeated these experiments with single-cell labeled data. The above analysis produced similar results, with source-localized MTPM increasing the SLR from 1.0 ± 0.03 to 3.9 ± 0.3, (p = 8E-12, paired T-test, n = 41 trials, mouse age 28 days, power used 600 mW). The sourced localized SLR was also significantly greater than the LED SLR of 1.16 ± 0.04 (p = 8E-11, Welch’s two sample T-test, n = 41 trials, mouse age 28 days). This indicated that the source localization does indeed reduce signal crosstalk by reassignment of scattered signal photons back to their emitted location. The smaller SLR seen in the single-cell labeled data is likely due to two factors. Firstly, the extracellular ROI used in the analysis is smaller to eliminate effects from reconstruction artefacts as discussed in the previous section. As scattering is highly forward biased, the closer pixels are likely to contain a higher quantity of signal and increase the extracellular signal size. Secondly, the increase in peak signal due to source localization is much higher in the multi-cell labeling case, likely because in this case the reassignment of scattered photons not only increases signal photon collection but also reduces non signal-containing extracellular photon collection in the intracellular ROI.

### 3.3. Source localization’s impact on PSNR is labeling and frame rate dependent

**Fig. 7:**
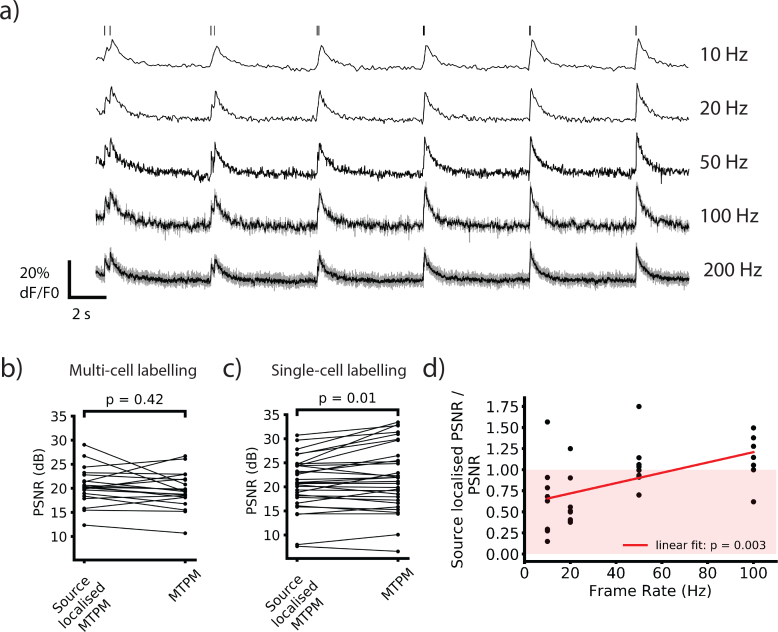
The effect of source localization on PSNR is labeling and frame rate dependent. a) Example single-cell labeled traces acquired at different frame rates. Traces are source localized for all but 200 Hz where no temporal oversampling or source localization was used. A binomial filter was applied to 100 and 200 Hz traces, and the raw trace is shown in gray. b & c) Comparisons of PSNR with and without source localization for all frame rates for multi-cell labeling (b) and single-cell labeling (c). Single-cell labeling shows a significant decrease in SNR when source localization is applied due to noise introduced in the deconvolution. This is not seen when imaging with densely labeled samples as the signal increase is much larger due to the reduction in non-signal-containing scattered light collected in the intracellular ROI. c) The ratio of source localized PSNR to non source-localized PSNR plotted against frame rate. This increases at higher frame rates as Poisson noise dominates over noise introduced in the deconvolution. Red area indicates worse performance for source localized videos.

The MTPM system captured neuronal calcium fluorescence transients at frame rates up to 200 Hz, with source-localization for frame rates up to 100 Hz (Fig. 7(a)

). Above 100 Hz the temporal oversampling required for source localization made the FOV with our camera prohibitively small. We wanted to ensure that source localization did not reduce the SNR increase of MTPM over single spot 2P by introducing additional noise to the recordings. To do this we compared the change in SNR between source localized and non source localized time courses. If the SNR is not decreased, then source localized MTPM increases signal through parallelization while reducing the drawbacks of wide-field detection in scattering samples. Source localization did not change the PSNR for multi-cell labeling significantly. The PSNR rose from 126 (21.0 dB) ± 26 to 162 (22.1 dB) ± 40 (p = 0.42, paired T-test, n = 20 trials, mouse age 36 days, power used 700 mW, Fig. 7(b)).

Applying source localization to the single-cell labeled traces significantly decreased the PSNR when looking at the pooled data from 443 (26.5 dB) ± 114 to 224 (23.5 dB) ± 48 (p = 0.01, paired T-test, n = 31, mouse age 28 days, power used 700 mW, Fig. 7(c)). The difference between the two results can be accounted for by a larger increase in the signal amplitude for the source localized MTPM when using multi-cell labeling.

With multi-cell labeling the source localized signal size is 2.29 ± 0.09 times greater than for non source localized traces, whereas the increase for single-cell labeling is 1.28 ± 0.012 (p = 5E-10, Welch’s T-test, n = 20, mouse age 36 days, power used 600 mW). As discussed in the previous section, source localization is more beneficial to multi-cell labeled samples. This is because the deconvolution process not only reassigns light scattered from the interior of the cell back inside, increasing the signal, it also reassigns light scattered from other bright areas into the interior of the cell back out again, reducing the background.

The decrease in PSNR due to source localization in single-cell labeled samples is inversely correlated with frame rate (Fig. 7(d)). When looking at frame rates above 50 Hz, there is no decrease in PSNR when using source localization. Below 50 Hz there is a significant decrease in PSNR. The peak signal increases at all frame rates when source localization is applied. The increase in the noise level during the source localization process is greater below 50 Hz. This suggests that the deconvolution is introducing a noise component that is frame rate independent. At low frame rates this is larger than the Poisson noise intrinsic to fluorescence imaging and makes up a large fraction of the total noise. Here the noise increase exceeds the increase in signal, reducing the PSNR. At high frame rates, Poisson noise dominates and the fraction of the total noise that the deconvolution contributes is small. The increase in signal amplitude then compensates, leaving an unchanged PSNR. As this decrease is only seen in singly-labeled cells, it will not be an issue for most typical use cases for multifocal imaging. If labeling is sufficiently sparse then high SNR one-photon excitation becomes a more viable imaging alternative. Multifocal imaging is best suited for situations where one-photon imaging would be impractical due to a large background fluorescence signal from non-target cells.

## 4. Conclusion

We have shown that exploiting the structured illumination inherent in multifocal two-photon microscopy can mitigate the effects of scattering that previously limited the extent of its application to mammalian brain imaging. The source localization process described here increases the image contrast at depth in scattering brain tissue, increasing the number of cells that can be sampled compared to regular MTPM. In brain slices, this can improve access to intact cells, synapses, and processes away from the cut surface. Future in vivo studies can also benefit as layer 2/3 of the mouse cortex lies at depths comparable to the 50% cutoffs for source localized MTPM. Although our implementation requires average powers known to heat brain tissue in vivo, future systems can avoid this problem with increased numerical apertures, higher laser pulse energies at decreased repetition rates, or dispersion compensation.

Source localization also reduces the mixing of signals from adjacent neuronal structures due to scattering. This is essential when neuronal activity is visualized by voltage indicators, as low amplitude fluctuations in their time course contain information about sub-threshold membrane potential dynamics. Their time course also cannot be modeled simply by a convolution of AP times with a characteristic pulse shape. Avoiding aliasing of this signal requires increasing the imaging frame rate.

Source localization does not decrease the PSNR of functional traces for densely labeled samples. The increase in brightness, which would usually increase PSNR, is offset by noise amplification during the deconvolution process. This could be mitigated by more sophisticated deconvolution algorithms, such as damped Richardson-Lucy [38]. The quality of the deconvolution could also be improved by refining the PSF estimate, or using blind deconvolution to adjust the PSF to each individual imaging location [39].

As the source localization process requires temporally oversampling the image series, currently the FOV height is limited by the camera speed and the oversampling factor for source-localized MTPM. The FOV can therefore be increased with faster cameras or a reduction in the temporal oversampling factor, although this would likely reduce the deconvolution efficacy, as discussed in section 3.1. A second limitation is the computational cost of Richardson-Lucy deconvolution. This is currently around 0.8 s per frame for an 391 × 80 μm FOV, leading to long computation times for high frame rate or long imaging sessions. Both the deconvolution itself and individual frame processing are parallelizable and so can improve with GPU or cluster based processing.

MTPM fills a gap between the commonly used imaging modalities of wide-field LED imaging and 2PLSM. MTPM achieves a speed and SNR closer to that of wide-field one-photon imaging without the large, out-of-focus background which swamps single-cell fluorescent transients and precludes cellular resolution population imaging. Source localized MTPM increases the scope of MTPM by mitigating its vulnerability to scattering. Source localized MTPM could prove particularly useful for two-photon voltage imaging with genetically encoded voltage indicators, which has so far struggled to achieve widespread use due to fast indicator temporal dynamics and small fluorescent responses.
